# General service and child immunization-specific readiness assessment of healthcare facilities in two selected divisions in Bangladesh

**DOI:** 10.1186/s12913-018-2858-7

**Published:** 2018-01-25

**Authors:** Md. Shajedur Rahman Shawon, Gourab Adhikary, Md. Wazed Ali, Md. Shamsuzzaman, Shahabuddin Ahmed, Nurul Alam, Katya A. Shackelford, Alexander Woldeab, Stephen S. Lim, Aubrey Levine, Emmanuela Gakidou, Md. Jasim Uddin

**Affiliations:** 1 International Centre for Diarrhoeal Disease Research, (icddr,b), Mohakhali, Dhaka, 1212 Bangladesh; 2 Nuffield Department of Population Health, University of Oxford, Oxford, OX3 7LF UK; 3 Expanded Programme on Immunisation, Directorate General of Health Services (DGHS), Mohakhali, Dhaka, 1212 Bangladesh; 4 Institute for Health Matrices and Evaluation (IHME), University of Washington, Seattle, USA

**Keywords:** Bangladesh, Healthcare facilities, Service readiness, Health service research, Healthcare system, Immunization, PCV, IPV

## Abstract

**Background:**

Service readiness of health facilities is an integral part of providing comprehensive quality healthcare to the community. Comprehensive assessment of general and service-specific (i.e. child immunization) readiness will help to identify the bottlenecks in healthcare service delivery and gaps in equitable service provision. Assessing healthcare facilities readiness also helps in optimal policymaking and resource allocation.

**Methods:**

A health facility survey was conducted between March 2015 and December 2015 in two purposively selected divisions in Bangladesh; i.e. Rajshahi division (high performing) and Sylhet division (low performing). A total of 123 health facilities were randomly selected from different levels of service, both public and private, with variation in sizes and patient loads from the list of facilities. Data on various aspects of healthcare facility were collected by interviewing key personnel. General service and child immunization specific service readiness were assessed using the Service Availability and Readiness Assessment (SARA) manual developed by World Health Organization (WHO). The analyses were stratified by division and level of healthcare facilities.

**Results:**

The general service readiness index for pharmacies, community clinics, primary care facilities and higher care facilities were 40.6%, 60.5%, 59.8% and 69.5%, respectively in Rajshahi division and 44.3%, 57.8%, 57.5% and 73.4%, respectively in Sylhet division. Facilities at all levels had the highest scores for basic equipment (ranged between 51.7% and 93.7%) and the lowest scores for diagnostic capacity (ranged between 0.0% and 53.7%). Though facilities with vaccine storage capacity had very high levels of service readiness for child immunization, facilities without vaccine storage capacity lacked availability of many tracer items. Regarding readiness for newly introduced pneumococcal conjugate vaccine (PCV) and inactivated polio vaccine (IPV), most of the surveyed facilities reported lack of sufficient funding and resources (antigen) for training programs.

**Conclusions:**

Our study suggested that health facilities suffered from lack of readiness in various aspects, most notably in diagnostic capacity. Conversely, with very few challenges, nearly all the health facilities designated to provide immunization services were ready to deliver routine childhood immunization services as well as newly introduced PCV and IPV.

## Background

The overall health status of Bangladeshi population has been continually improving over the past few decades. They have experienced a major decline (i.e. annual rate of 5.6%) in maternal mortality ratio (MMR) from 322 deaths to 194 deaths per 100,000 live births between 1998 and 2001 and 2007–10 [1]. Furthermore, Bangladesh has already achieved the Millennium Development Goal (MDG) 4 target, under-5 mortality rate of 48 deaths per 1000 live births [2]. However, due to simultaneous demographic and epidemiological transitions coupled with rapid urbanization, Bangladesh has been suffering from double burden of disease – emergence of non-communicable disease and re-emergence of various communicable diseases like pneumonia, diarrhoea, malaria, tuberculosis [3]. Moreover, the major challenges of Bangladesh public health system are lack of access, poor quality of care, lack of comprehensiveness and inequitable distribution of resources [4].

To improve the health outcomes by increasing the supply of human and financial resources in effective and efficient manner, assessing health system readiness for provision of general and specific services is crucial. Furthermore, such comprehensive assessment of readiness will help to assess the health system performance by identifying the bottlenecks in healthcare service delivery and gaps in equitable service provision throughout the country. Assessing healthcare facilities readiness helps in optimal policymaking and resource allocation. However, due to the multidimensionality of health system functions, comprehensive and detailed assessment seldom occurs. As a result, information about infrastructure, healthcare providers, diagnostic capacity, equipment and drugs are often missing or lack quality [5].

Access to quality healthcare includes several dimensions, such as availability, affordability and acceptability [6, 7]. However, provision of quality services also depends on service readiness. The term “readiness” has been defined as achieving and maintaining a state of preparedness in the facility to provide comprehensive quality care to the patients indicated by availability of trained staff, infrastructure, guidelines, essential drugs, medical commodities and diagnostic capacity [8]. Though it cannot guarantee the actual delivery of quality services, service readiness is one of the indispensable aspect for provision of comprehensive quality healthcare [8]. Since existing literature have focused on service availability and readiness in fragmented approaches leading to various information gaps [9], World Health Organization (WHO) along with its global partners have developed a standard tools like Service Availability and Readiness Assessment (SARA) which will allow capturing facility readiness in a comprehensive manner and therefore, fill the critical information gaps in evaluating health systems strengthening programs [8].

Bangladesh has been gaining tremendous success in achieving high level of immunization coverage (for example, the current full immunization coverage is 84%) since the advent of Expanded Program on Immunization (EPI) in 1979 [2]. However, still there are significant differences in coverage among the administrative divisions, ranging between 61% and 90% [2]. While many of the previous studies have looked at the demand side issues of existing gaps in immunization program [10–13], there is still paucity in exploring how supply side i.e. facility-based factors may interact with patient perspectives to hinder optimal delivery of immunization services. Thus, assessing immunization specific readiness in low and high performing areas would provide insights for improving immunization service delivery and related population health outcomes.

In accordance with the ongoing Polio Eradication & Endgame Strategic Plan 2013–2018, Bangladesh has introduced inactivated polio vaccine (IPV) into its regular national immunization schedule in the year 2015 in collaboration with Gavi, the Vaccine Alliance; UNICEF; WHO and the Global Polio Eradication Initiative (GPEI) partners [14]. Moreover, pneumococcal conjugate vaccine (PCV) has also been introduced at the same timeframe to combat pneumonia, a major cause of under-5 children mortality. With already high immunization coverage in Bangladesh, the effectiveness of introducing these two vaccines into national immunization program highly depends on their service availability and readiness. Thus, in this study, we also assessed service readiness for PCV and IPV introduction in terms of training, funding and vaccine availability.

In this study, we assessed general service and child immunization-specific healthcare facility readiness in two selected administrative divisions in Bangladesh, by facility levels. Additionally, we appraised the readiness for recent introduction of PCV and IPV vaccines into regular immunization program.

## Methods

### Study design and settings

This was a cross-sectional study conducted between March and August in 2015. The present study used the data from the assessment of access, bottlenecks, costs and equity in health sector of Bangladesh (ABCE) study. The ABCE study had three components: i) health facility survey; ii) patient exit interview; and iii) District Civil Surgeon Office or City Corporation office survey. We assessed both general service and child immunization-specific readiness from the health facility survey data. This study was approved by the institutional review board at University of Washington, Seattle, USA and International Centre for Diarrhoeal Disease Research, Bangladesh (icddr,b).

Bangladesh is divided into seven administrative divisions which are sub-divided into 64 districts, and further into 485 upazilas (sub-districts). This study was conducted in two divisions, namely Rajshahi and Sylhet. Rajshahi division has 8 districts with a total area of 18,154 km^2^ while Sylhet division consists of 4 districts and has total area of 12,558 km^2^. These two divisions were purposely selected because there are large regional disparities in health indicators between them. For example, infant mortality rate was 33 and 55 per 1000 live births in Rajshahi and Sylhet, respectively. Moreover, Rajshahi division has better immunization coverage (83.6% vs. 61.1%) and contraceptive prevalence rate (67.3% vs. 44.8%) than Sylhet [2, 15]. Public health system in Bangladesh is divided into three tiers, for example, primary, secondary and tertiary care level. Community clinics, union health and family welfare centers, union sub-centers and upazilla health complexes are included in the primary care level. District hospitals belong to the secondary care level and tertiary care level includes medical college hospitals and specialized hospitals. In private sector, Bangladesh has pharmacies in the primary level whereas the secondary and tertiary levels include non-government organization (NGO) clinics & hospitals as well as private clinics & hospitals.

### Sample size and sampling of health facilities

We calculated the sample size using the sampling manual for facility surveys developed by MEASURE Evaluation [16] where we set the anticipated proportion of facilities with the attribute of interest (p) as 50%; the design effect as 1.2 and the width of confidence interval for key estimates at p ± 0.2p. This information yielded a sample size of 115 facilities; but we anticipated 10% non-response rate, therefore we planned to survey 126 facilities. Due to budget and field constraints, we divided this sample into our two selected divisions. From the existing database of Directorate General of Health Services (DGHS), we collected basic information about all the primary, secondary and tertiary level health facilities, both public and private, for the two selected divisions. From Rajshahi division one rural area (Joypurhat district) and one urban area (Rajshahi City Corporation) were purposively selected. Joypurhat district has a total of 166 healthcare facilities (both public and private), majority of which are community clinics (66.3%). On the other hand, Rajshahi City Corporation has 63 healthcare facilities in total. From Sylhet division, one rural area (e.g. Sylhet district) and one urban area (e.g. Sylhet City Corporation) were purposively selected. There are 314 health facilities of various levels (community clinics 77.4%) in Sylhet district and 54 health facilities in Sylhet City Corporation. From this list, we then randomly sampled health facilities from different levels of service, both public and private, with variation in sizes and patient loads from the list of facilities. Finally, we ended up having 12 pharmacies, 12 community clinics, 15 primary care facilities and 22 higher care facilities in each division. The non-response rate was less than 3%.

### Data collection

Data was collected through an electronic structured questionnaire. ABCE is a multi-country research project undertaken by Institute for Health Metrics and Evaluation (IHME). Therefore, the questionnaire was developed by experts from IHME with consultation from local stakeholders and health system researchers [17]. The questionnaire had eight modules, namely inputs and finance; facilities management and infrastructure; laboratory consumables, equipment and facility capacity; pharmaceuticals; medical consumables, equipment and facility capacity; ABCE facility outputs; Gavi vaccine module and finally temperature loggers. The questionnaire was translated into Bengali (the local language) and then back-translated to English to see the consistency in the translation process. Pre-test of the questionnaire was carried out and required amendments were done based on the feedback received from the pre-test. The research assistants were trained and given laptops containing the electronic structured questionnaire. They first communicated with the facility administrator to schedule an appropriate visit day and time and then collected related information from the key personnel for specific modules. The data for present study came from modules on facility management and infrastructure; laboratory consumables & equipment; pharmaceuticals; medical consumables & equipment, Gavi vaccine module and temperature logger. Data for these modules was mainly collected by interviewing key personnel, however direct observation on facility infrastructure and management was done to triangulate with the data collected from interviews.

### Data analyses

We analyzed the data following the WHO Service Availability and Readiness Assessment (SARA) manual [8] to assess general service readiness of the surveyed facilities. The SARA guideline looked into five domains of general service readiness, for example, basic amenities, basic equipment, standard precautions for infection prevention, diagnostic capacity and essential medicines. Since the essential medicine list provided by the SARA manual is different from that of Bangladesh government, we used the local list of essential medicines to assess readiness in that domain. Score for each domain was calculated based on the mean availability of tracer items as percentage within that domain. Finally, mean of all the five domain scores were calculated and expressed as general service readiness index [8].

For general service readiness assessment, we stratified all surveyed facilities into four groups. Even though the community clinics and pharmacies belong to the primary care level in Bangladesh health system, we analyzed them separately from other primary care facilities due to the obvious differences in their structure and scope of services. We included union health and family welfare centers, union sub-centers and upazilla health complex in the primary care facility group whereas district hospitals, NGO clinics and hospitals, private clinics and hospitals and medical college hospitals were grouped into higher care facilities.

For child immunization specific readiness assessment we also followed SARA guidelines. Here, we categorized the eligible healthcare facilities into two groups: facilities with vaccine storage capacity and facilities without vaccine storage capacity. Since SARA guideline did not mention about domain-specific scoring system for child immunization, we did not calculate domain scores for readiness assessment. Additionally, we also assessed the service readiness for PCV and IPV vaccines, stratified by facility level, based on the available facility data for these two vaccines.

All the results are summarized and presented as frequencies and percentages by facility level for categorical variables and mean and standard deviation for continuous variables. All analyses were conducted using Stata version 14.1.

## Results

### Characteristics of healthcare facilities

The present study included a sample of 123 healthcare facilities (i.e. 61 from Rajshahi division and 62 form Sylhet division) with a total of 10,253 staff working at different levels. The sample included equal number of pharmacies, community clinics, primary care facilities and higher care facilities from each selected division. While all included pharmacies were private and all community clinics were public facilities, 80% of primary care facilities and 18.2% of higher care facilities were public. All community clinics and most of the primary care facilities (87%) were in the rural area. Among the human resources, nurses represented the majority (*n* = 1802; 17.6%), followed by the doctors (*n* = 1165; 11.4%).

### General service readiness

**Fig. 1:**
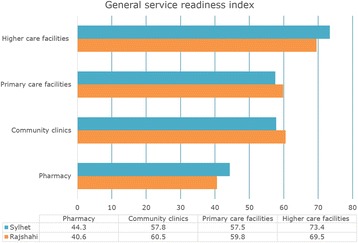
General service readiness index by healthcare facility levels in a high-performing division (Rajshahi) and a low-performing division (Sylhet) in Bangladesh

The general service readiness index was 40.6%, 60.5%, 59.8% and 69.5% for pharmacies, community clinics, primary care facilities and higher care facilities, respectively in Rajshahi division. In Sylhet division, the general service readiness indexes for pharmacies, community clinics, primary care facilities and higher care facilities were 44.3%, 57.8%, 57.5% and 73.4%, respectively (Fig. 1). Despite having differences in overall service readiness indexes by facility levels between Rajshahi and Sylhet division, the corresponding standard errors overlapped indicating no statistically significant differences.

Table 1 presents the results for four domains of general service readiness, namely basic amenities, basic equipment, standard precautions for infection prevention, and diagnostic capacity by healthcare facility types and by divisions. For all these four domains, higher care facilities had the highest domain scores followed by primary care facilities, community clinics and pharmacies. For essential medicines domain, community clinics had the highest score followed by pharmacies and higher care facilities had the lowest score (Table 2). The mean availability of items was lowest in the diagnostic capacity domain (between 0% and 53%) and highest in the basic equipment domain (between 52% and 94%). In domains like basic amenities, basic equipment, standard precautions for infection prevention and diagnostic capacity, healthcare facilities in Rajshahi division outperformed their counterparts in Sylhet division (Table 1). However, for essential medicines, primary and higher care facilities in Sylhet showed higher availability than that of Rajshahi division. Community clinics and pharmacies in Rajshahi division had higher availability of essential medicines in compared to community clinics and pharmacies in Sylhet division (Table 2).

**Table 1:** Assessment of general service readiness by healthcare facility levels in a selected high-performing division (Rajshahi) and a low-performing division (Sylhet) in Bangladesh

Variable	Rajshahi (*N* = 61)				Sylhet (*N* = 62)			
	Pharmacy	Community clinic	Primary care facilities	Higher care facilities	Pharmacy	Community clinic	Primary care facilities	Higher care facilities
General characteristics								
Number of facilities [n (% of total)]	12 (19.4)	12 (19.4)	15 (24.2)	22 (35.5)	12 (19.7)	13 (21.3)	15 (24.6)	22 (36.1)
Public facilities [n (%)]	0 (0.0)	12 (100.0)	12 (80.0)	4 (18.2)	0 (0.0)	13 (100.0)	12 (80.0)	4 (18.2)
Area [n (%)]								
Rural	6 (50.0)	12 (100.0)	13 (86.7)	10 (45.5)	6 (50.0)	13 (100.0)	13 (86.7)	11 (50.0)
Urban	6 (50.0)	0 (0.0)	2 (13.3)	12 (54.5)	6 (50.0)	0 (0.0)	2 (13.3)	11 (50.0)
Duration of service, in years [Mean (SD)]	4 (2.9)	13 (3.3)	28 (15.1)	16 (16.1)	9 (10.4)	10 (5.6)	28 (14.1)	20 (17.4)
Basic amenities								
Power [n (%)]	4 (33.3)	5 (41.7)	12 (80.0)	20 (90.9)	7 (58.3)	0 (0.0)	4 (26.7)	18 (81.8)
Improved water source within 500 m of facility [n (%)]	11 (91.7)	12 (100.0)	15 (100.0)	22 (100.0)	9 (75.0)	10 (76.9)	14 (93.3)	22 (100.0)
Room with auditory and visual privacy for patient consultations [n (%)]	1 (8.3)	9 (75.0)	11 (73.3)	22 (100.0)	4 (33.3)	12 (92.3)	12 (80.0)	21 (95.5)
Access to adequate sanitation facilities for clients [n (%)]	1 (8.3)	12 (100.0)	15 (100.0)	22 (100.0)	3 (25.0)	12 (92.3)	15 (100.0)	22 (100.0)
Communication equipment (phone or SW radio) [n (%)]	12 (100.0)	0 (0.0)	6 (40.0)	22 (100.0)	10 (83.3)	1 (7.7)	4 (26.7)	20 (90.9)
Facility has access to computer [n (%)]	0 (0.0)	11 (91.7)	11 (73.3)	18 (81.8)	3 (25.0)	11 (84.6)	10 (66.7)	17 (77.3)
Emergency transportation [n (%)]	3 (25.0)	12 (100.0)	5 (33.3)	9 (40.9)	1 (8.3)	10 (76.9)	4 (26.7)	15 (68.2)
Domain score (Mean availability of items as percentage) [Mean (SE)]	38.1 (15.5)	72.6 (14.5)	71.4 (9.9)	87.7 (8.2)	44.0 (10.7)	61.5 (15.1)	60.0 (12.4)	87.7 (4.6)
Basic equipment								
Adult scale [n (%)]	3 (25.0)	11 (91.7)	15 (100.0)	21 (95.5)	2 (16.7)	12 (92.3)	9 (60.0)	19 (86.4)
Child scale [n (%)]	0 (0.0)	4 (33.3)	10 (66.7)	16 (72.7)	0 (0.0)	6 (46.2)	11 (73.3)	19 (86.4)
Thermometer [n (%)]	10 (83.3)	12 (100.0)	13 (86.7)	22 (100.0)	10 (83.3)	12 (92.3)	12 (80.0)	21 (95.5)
Stethoscope [n (%)]	9 (75.0)	12 (100.0)	15 (100.0)	22 (100.0)	6 (50.0)	10 (76.9)	14 (93.3)	22 (100.0)
Blood pressure apparatus [n (%)]	9 (75.0)	10 (83.3)	15 (100.0)	22 (100.0)	9 (75.0)	9 (69.2)	14 (93.3)	22 (100.0)
Domain score (Mean availability of items as percentage) [Mean (SE)]	51.7 (16.5)	81.7 (12.5)	90.7 (6.5)	93.6 (5.3)	75.4 (8.6)	75.4 (8.6)	80.0 (6.3)	93.7 (3.1)
Standard precautions for infection prevention								
Safe final disposal of sharps [n (%)]	6 (50.0)	11 (91.7)	15 (100.0)	22 (100.0)	6 (50.0)	13 (100.0)	12 (80.0)	21 (95.5)
Safe final disposal of infectious wastes [n (%)]	6 (50.0)	11 (91.7)	13 (86.7)	22 (100.0)	6 (50.0)	13 (100.0)	14 (93.3)	21 (95.5)
Appropriate storage of sharps waste [n (%)]	0 (0.0)	0 (0.0)	5 (33.3)	21 (95.5)	0 (0.0)	2 (15.4)	7 (46.7)	20 (90.9)
Domain score (Mean availability of items as percentage) [Mean (SE)]	33.3 (16.7)	61.1 (30.6)	73.3 (20.4)	98.5 (1.5)	33.3 (16.7)	71.8 (28.2)	73.3 (13.9)	94.0 (1.5)
Diagnostic capacity								
Hemoglobin [n (%)]	0 (0.0)	0 (0.0)	5 (33.3)	14 (63.6)	0 (0.0)	0 (0.0)	6 (40.0)	11 (50.0)
Blood glucose [n (%)]	6 (50.0)	0 (0.0)	4 (26.7)	15 (68.2)	4 (33.3)	0 (0.0)	5 (33.3)	18 (81.8)
Malaria diagnostic capacity [n (%)]	0 (0.0)	0 (0.0)	3 (20.0)	6 (27.3)	0 (0.0)	0 (0.0)	1 (6.7)	10 (45.5)
Urine dipstick- protein, glucose [n (%)]	0 (0.0)	0 (0.0)	5 (33.3)	15 (68.2)	0 (0.0)	0 (0.0)	6 (40.0)	10 (45.5)
Syphilis rapid test [n (%)]	0 (0.0)	0 (0.0)	0 (0.0)	8 (36.4)	0 (0.0)	0 (0.0)	0 (0.0)	10 (45.5)
Domain score (Mean availability of items as percentage) [Mean (SE)]	10.0 (10.0)	0.0 (0.0)	22.7 (6.2)	52.7 (8.7)	6.7 (6.7)	0.0 (0.0)	24.0 (8.6)	53.6 (7.1)

*N* Total number of facilities, *n* Number of facilities in specific category, *SD* Standard Deviation, *SE* Standard error

**Table 2:** Availability of essential medicines by healthcare facility levels in a selected high-performing division (Rajshahi) and a low-performing division (Sylhet) in Bangladesh

Variable	Rajshahi (*N* = 61)				Sylhet (*N* = 62)			
	Pharmacy	Community clinic	Primary care facilities	Higher care facilities	Pharmacy	Community clinic	Primary care facilities	Higher care facilities
Albendazole [n (%)]	10 (83.3)	12 (100.0)	12 (80.0)	3 (13.6)	9 (75.0)	13 (100.0)	14 (93.3)	10 (45.5)
Amoxicillin Cap [n (%)]	11 (91.7)	11 (91.7)	6 (40.0)	4 (18.2)	10 (83.3)	12 (92.3)	6 (40.0)	11 (50.0)
Amoxicillin Syrup/Pediatric suspension [n (%)]	11 (91.7)	12 (100.0)	7 (46.7)	2 (9.1)	8 (66.7)	12 (92.3)	10 (66.7)	7 (31.8)
Antacid [n (%)]	11 (91.7)	12 (100.0)	11 (73.3)	5 (22.7)	10 (83.3)	13 (100.0)	15 (100.0)	10 (45.5)
Benzoic Acid and Salicylic Acid Ointment [n (%)]	6 (50.0)	12 (100.0)	6 (40.0)	4 (18.2)	3 (25.0)	12 (92.3)	3 (20.0)	3 (13.6)
Benzyl Benzoate Lotion Application [n(%)]	1 (8.3)	12 (100.0)	4 (26.7)	1 (4.5)	3 (25.0)	11 (84.6)	4 (26.7)	3 (13.6)
Calcium Lactated Tab [n (%)]	9 (75.0)	12 (100.0)	3 (20.0)	4 (18.2)	9 (75.0)	12 (92.3)	5 (33.3)	10 (45.5)
Chloramphenicol Eye/Ear/Ointment/Drops [n (%)]	8 (66.7)	12 (100.0)	8 (53.3)	4 (18.2)	11 (91.7)	12 (92.3)	10 (66.7)	9 (40.9)
Chlorpheniramine Maleate Tablet [n (%)]	4 (33.3)	12 (100.0)	8 (53.3)	4 (18.2)	6 (50.0)	10 (76.9)	5 (33.3)	6 (27.3)
Cotriomoxazole Tab [n (%)]	7 (58.3)	12 (100.0)	10 (66.7)	2 (9.1)	6 (50.0)	10 (76.9)	14 (93.3)	5 (22.7)
Doxycycline [n (%)]	12 (100.0)	12 (100.0)	7 (46.7)	3 (13.6)	10 (83.3)	12 (92.3)	11 (73.3)	10 (45.5)
Ferrous Sulphate Tablet/ Syrup [n (%)]	6 (50.0)	0 (0.0)	4 (26.7)	4 (18.2)	7 (58.3)	0 (0.0)	8 (53.3)	7 (31.8)
Folic Acid Tablet [n (%)]	11 (91.7)	9 (75.0)	11 (73.3)	2 (9.1)	9 (75.0)	11 (84.6)	10 (66.7)	9 (40.9)
Gentian Violet Topical Solution [n (%)]	1 (8.3)	12 (100.0)	0 (0.0)	1 (4.5)	4 (33.3)	11 (84.6)	0 (0.0)	2 (9.1)
Hyoscine ButyIbromide [n (%)]	8 (66.7)	12 (100.0)	4 (26.7)	3 (13.6)	2 (16.7)	13 (100.0)	2 (13.3)	9 (40.9)
Mebendazole Tablet [n (%)]	6 (50.0)	0 (0.0)	0 (0.0)	5 (22.7)	8 (66.7)	0 (0.0)	4 (26.7)	14 (63.6)
Neomycin Bacitracin skin ointment [n (%)]	7 (58.3)	12 (100.0)	4 (26.7)	2 (9.1)	8 (66.7)	12 (92.3)	3 (20.0)	6 (27.3)
ORS (Oral Rehydration Salt) [n (%)]	12 (100.0)	12 (100.0)	7 (46.7)	5 (22.7)	10 (83.3)	13 (100.0)	10 (66.7)	12 (54.5)
Paracetamol suspension/syrup [n (%)]	12 (100.0)	12 (100.0)	9 (60.0)	4 (18.2)	8 (66.7)	11 (84.6)	7 (46.7)	10 (45.5)
Paracetamol Tab Tablet/Elixir [n (%)]	11 (91.7)	12 (100.0)	11 (73.3)	5 (22.7)	10 (83.3)	12 (92.3)	15 (100.0)	14 (63.6)
Benzathine Penicillin [n (%)]	2 (16.7)	0 (0.0)	1 (6.7)	1 (4.5)	3 (25.0)	0 (0.0)	1 (6.7)	4 (18.2)
Salbutamol syrup [n (%)]	10 (83.3)	12 (100.0)	2 (13.3)	4 (18.2)	5 (41.7)	11 (84.6)	2 (13.3)	8 (36.4)
Salbutamol Tablet/Elixir/Inhaler/Injection [n (%)]	10 (83.3)	12 (100.0)	7 (46.7)	5 (22.7)	7 (58.3)	11 (84.6)	12 (80.0)	12 (54.5)
Vitamin A Cap (200,000 I.U.) [n (%)]	9 (75.0)	12 (100.0)	3 (20.0)	1 (4.5)	7 (58.3)	13 (100.0)	5 (33.3)	4 (18.2)
Vitamin B- Complex Tablet/Multi-vitamin D drops 15 ml [n (%)]	12 (100.0)	12 (100.0)	11 (73.3)	4 (18.2)	10 (83.3)	13 (100.0)	14 (93.3)	12 (54.5)
Zinc [n (%)]	11 (91.7)	12 (100.0)	3 (20.0)	4 (18.2)	10 (83.3)	11 (84.6)	6 (40.0)	10 (45.5)
Domain score (Mean availability of items as percentage) [Mean (SE)]	69.9 (5.6)	87.2 (6.4)	40.8 (4.7)	15.0 (1.2)	61.9 (4.4)	80.2 (5.9)	50.3 (6.1)	37.9 (3.0)

*N* Total number of facilities, *n* Number of facilities in specific category, *SE* Standard error

### Child immunization service readiness

In our sample, 59 facilities (28 from Rajshahi and 31 from Sylhet) were involved in routine child immunization services. Among them only 9 had vaccine storage service available and all of them were public facilities. Among facilities offering immunization services without any vaccine storage, 92% and 81% were public facilities in Rajshahi and Sylhet division, respectively. There was no vaccination center in any tertiary hospitals, private clinics or pharmacies. Most of these health facilities, irrespective of vaccine storage capacity, were in the rural areas (Table 2). Facilities with vaccine storage facilities had higher mean service days per month both in Rajshahi division (7 days; SD 5.0) and Sylhet division (21 days; SD 7.3). Almost all these facilities had outreach services available.

Among the facilities with vaccine storage capacity in the high performing division (Rajshahi), most of the tracer items for routine child immunization were available for all the facilities. For similar facilities in Sylhet division, few items in the equipment domain as well as EPI guidelines were unavailable for one or more facilities. Though there was regular supply and availability for BCG, Measles & Rubella, Penta and Polio vaccines in these facilities, availability of PCV and IPV vaccines fell short. In facilities without vaccine storage capacity, auto-disposable syringe was available, but only a few of them had sharp container to dispose sharp materials. There was no stock-out of vaccines except IPV in Rajshahi division in the last quarter preceding the survey (Table 3).

**Table 3:** Assessment of service readiness for routine child immunization according to healthcare facility levels in the two selected divisions of Bangladesh

Variables	Rajshahi division (*N* = 28)		Sylhet division (*N* = 31)	
	Facilities with vaccine storage	Facilities without vaccine storage	Facilities with vaccine storage	Facilities without vaccine storage
General characteristics				
Number of facilities [n]	4	24	5	26
Public facility [n (%)]	4 (100.0)	22 (91.7)	5 (100.0)	21 (80.8)
Area [n (%)]				
Rural	4 (100.0)	22 (91.7)	4 (80.0)	23 (88.5)
Urban	0 (0.0)	2 (8.3)	1 (20.0)	3 (11.5)
Vaccines and injection supplies are bundled [n (%)]				
No	0 (0.0)	0 (0.0)	0 (0.0)	4 (15.4)
Yes, only pick up	4 (100.0)	24 (100.0)	2 (40.0)	7 (26.9)
Yes, both	0 (0.0)	0 (0.0)	3 (60.0)	14 (53.8)
Service days per month [Mean (SD)]	7 (5.0)	4 (7.8)	21 (7.3)	2 (2.6)
Hours of service on a typical day [Mean (SD)]	4 (2.5)	5 (0.6)	6 (0.5)	6 (0.8)
Staffs involved in vaccination [Mean (SD)]	2 (1.0)	2 (0.4)	1 (0.5)	2 (0.6)
Outreach services available [n (%)]	4 (100.0)	0 (0.0)	4 (80.0)	0 (0.0)
Staff and training				
Guidelines for EPI %	4 (100.0)	0 (0.0)	2 (40.0)	0 (0.0)
Staff trained in EPI %	4 (100.0)	24 (100.0)	5 (100.0)	25 (96.2)
Equipment				
Cold box/vaccine carrier with ice packs [n (%)]	4 (100.0)	0 (0.0)	5 (100.0)	0 (0.0)
Refrigerator [n (%)]	4 (100.0)	0 (0.0)	5 (100.0)	0 (0.0)
Sharp container [n (%)]	1 (25.0)	2 (8.3)	1 (20.0)	4 (15.4)
Single use- standard disposable or auto-disable syringes [n (%)]	4 (100.0)	24 (100.0)	5 (100.0)	25 (96.2)
Continuous temperature monitoring device in refrigerator [n (%)]	4 (100.0)	0 (0.0)	4 (80.0)	0 (0.0)
Energy source and power supply for vaccine refrigerator [n (%)]	4 (100.0)	0 (0.0)	4 (80.0)	0 (0.0)
Immunization cards [n (%)]	4 (100.0)	22 (91.7)	4 (80.0)	18 (69.2)
Medicines and commodities				
Measles & Rubella vaccine [n (%)]	4 (100.0)	4 (16.7)	5 (100.0)	1 (3.8)
Penta (DPT + Hib + HepB) vaccine [n (%)]	4 (100.0)	4 (16.7)	5 (100.0)	1 (3.8)
Polio vaccine [n (%)]	4 (100.0)	4 (16.7)	5 (100.0)	1 (3.8)
BCG vaccine [n (%)]	4 (100.0)	4 (16.7)	5 (100.0)	0 (0.0)
PCV [n (%)]	4 (100.0)	3 (12.5)	3 (60.0)	1 (3.8)
IPV [n (%)]	3 (75.0)	2 (8.3)	5 (100.0)	1 (3.8)
Stock-outs in the past 3 months				
Measles & Rubella vaccine [n (%)]	0(0.0)	0(0.0)	0(0.0)	0(0.0)
Penta (DPT + Hib + HepB) vaccine [n (%)]	0(0.0)	0(0.0)	0(0.0)	0(0.0)
Polio vaccine [n (%)]	0(0.0)	0(0.0)	1(20.0)	0(0.0)
BCG vaccine [n (%)]	0(0.0)	0(0.0)	0(0.0)	0(0.0)
PCV [n (%)]	0(0.0)	0(0.0)	0(0.0)	0(0.0)
IPV [n (%)]	3(75.0)	0(0.0)	0(0.0)	0(0.0)

*BCG* Bacillus Calmette-Guérin, *EPI* Expanded Program on Immunization, *IPV* Inactivated Polio Vaccine, *PCV* Pneumococcal Conjugate Vaccine, *SD* Standard deviation

### Readiness for PCV and IPV introduction

Since Bangladesh has introduced PCV and IPV in 2015, we also assessed readiness for PCV and IPV introduction into the regular immunization program (Table 4). All the facilities with storage capacity and most of the facilities without storage capacity received official training for PCV and IPV immunization. Average number of staffs trained for both PCV and IPV in facilities with storage capacity was 72 and 114 in Rajshahi and Sylhet division, respectively. They lacked insufficient funding and antigen availability for training activities. Though the guidelines and posters for PCV were available in many facilities, these materials were not available for IPV.

**Table 4:** Assessment of service readiness for Pneumococcal conjugate vaccine (PCV) and PCV and Inactivated polio vaccine (IPV) implementation according to healthcare facility levels in the two selected divisions of Bangladesh

Variables	Rajshahi division (*N* = 28)		Sylhet division (*N* = 31)	
	Facilities with vaccine storage	Facilities without vaccine storage	Facilities with vaccine storage	Facilities without vaccine storage
Pneumococcal conjugate vaccine (PCV)				
Received official training about PCV [n (%)]	4 (100.0)	23 (95.8)	5 (100.0)	22 (84.6)
Total staff trained in PCV [Mean ± SD]	72 (66.1)	4 (1.2)	114 (64.3)	4 (5.5)
Antigen availability for PCV training [n (%)]	0 (0.0)	11 (45.8)	2 (40.0)	11 (42.3)
Sufficient fund for PCV training [n (%)]	2 (50.0)	0 (0.0)	2 (40.0)	1 (3.8)
Availability of PCV poster at the facility [n (%)]	2 (50.0)	3 (12.5)	4 (80.0)	13 (50.0)
Availability of PCV guidelines [n (%)]	3 (75.0)	0 (0.0)	4 (80.0)	0 (0.0)
Inactivated polio vaccine (IPV)				
Received official training about IPV [n (%)]	4 (100.0)	23 (95.8)	5 (100.0)	22 (84.6)
Staff trained in IPV [Mean ± SD]	72 (67.0)	4 (1.2)	114 (64.8)	3 (4.1)
Antigen availability for PCV training [n (%)]	0 (0.0)	10 (41.7)	3 (60.0)	9 (34.6)
Sufficient fund for IPV training [n (%)]	1 (25.0)	0 (0.0)	3 (60.0)	1 (3.8)
Availability of IPV poster at the facility [n (%)]	0 (0.0)	0 (0.0)	0 (0.0)	11 (42.3)
Availability of IPV guidelines [n (%)]	–	–	–	–

*IPV* Inactivated Polio Vaccine, *PCV* Pneumococcal Conjugate Vaccine, *SD* Standard deviation

## Discussion

The major findings of this study are – i) all healthcare facilities, except the pharmacies, in the two selected divisions in Bangladesh had moderate to high level of general service readiness with no obvious differences between the high and low performing division; ii) the facilities, irrespective of their levels, had the highest scores for basic equipment domain and the lowest scores for diagnostic capacity domain; iii) assessment of facilities providing child immunization services showed very high levels of service readiness among the facilities with vaccine storage in both the selected divisions whereas facilities without vaccine storage capacity suffered badly from poor service readiness; iv) the facilities offering child immunization services suffers particularly from inadequate funding and vaccine resources for training programs on newly introduced PCV and IPV vaccines.

Though Bangladesh has a strong layout of public health service delivery system with great emphasis on primary healthcare, general user perception of the quality of healthcare service delivery is not enlightening-encompassing complaints about the non-responsiveness of the healthcare providers, long waiting periods, absenteeism of the providers and poor quality of offered services [18]. Service availability and readiness is a precondition for providing comprehensive quality services to all segments of the population to ensure universal health coverage. Service readiness depends not only on presence of skilled providers but also on facility-level infrastructure, resources, medical equipment and diagnostic capacity. In this study, almost all the facilities were equipped with basic amenities and equipment to provide services. Continuous supply of electricity, provision of emergency transportation, laboratory and diagnostic equipment and management of sharp waste were some particular areas of concern and need to be further improved in all facilities.

Our study found major shortage of diagnostic capacity in most of the facilities, irrespective of facility level and/or facility type. Last nationwide health facility survey also found that basic diagnostic capacity in health facilities is low, for example, only 10% of the facilities had provision for hemoglobin test [9]. Such scarce of diagnostic tests limits the ability of healthcare providers to provide quality care. Healthcare providers pointed out inadequate infrastructure, supplies and trained technicians as barriers to inefficient diagnostic services [19]. It is pertinent to mention that, health system in Bangladesh is suffering from not only human resource shortage but also from poor skill mix. The current ratio of doctors to nurses to health technologists in Bangladesh is 1: 0.4: 0.24 – stark opposite of WHO recommended standards (i.e. doctors: nurses: technologists = 1: 3: 5) [4, 20].

Efficient and equitable distribution of medicines in the facilities is still a major challenge in public healthcare delivery system. Cockroft and colleagues, in a study based on three national community-based surveys, identified lack of/poor quality of medicines as one of the major cause of patient dissatisfaction in government health facilities [21]. Establishment and operationalization of 12,815 community clinics at ward level through “Revitalization of Community-based Healthcare Initiatives in Bangladesh” project has improved the availability of essential drugs at community level [2]. However, in a recent assessment of community clinics done by NIPORT [22], 39% of the users and 72% of the service providers complained about shortage of medicines. Less availability of medicines at various public facilities was also highlighted in the recent national health facility survey report [9].

Bangladesh has been maintaining high immunization coverage over the years through its intricate web of government facilities, private facilities and NGOs. National coverage for all vaccines was 84% in 2014 [2]. In our study sample, immunization service was available in 88% of all union-level facilities, 88% of sub-district level facilities and 92% of community clinics. We observed that most of the health facilities had adequate readiness for providing newly introduced vaccines, IPV and PCV. A few facilities experienced stock-out for IPV vaccine whereas supply of other vaccines, overall, was adequate and consistent. Though uninterrupted supply of electricity for vaccine storage was not possible in all the vaccine storage facilities, the use of ice-lined refrigerators helps to overcome the problem of irregular supply of electricity. Continuous temperature monitoring through temperature logger revealed that recommended temperature for vaccine storage (i.e. 2 to 8 degree Celsius) was maintained in all the facilities for nearly 100% of the time (data not shown).

Introduction of IPV and PCV would strengthen routine immunization program in Bangladesh, helping to maintain high coverage. PCV would reduce pneumonia incidence among under-5 children and contribute to increased child survival in a cost-effective manner [14]. In Bangladesh, childhood pneumonia is responsible for 22% of all child deaths [15]. On the other hand, Bangladesh government has introduced IPV into routine immunization activities with an effort to maintain its polio-free status. Administering IPV simultaneously with oral polio vaccine (OPV) would accelerate immunity and help to eradicate polio.

The major strength of our study is representation of health facilities from all strata, for example, urban vs. rural; public vs. private; primary care facilities vs. higher facilities. We also combined interview data with direct observation of the facility and record review to increase the validity of collected information. The use of objective criteria for assessment of service readiness was also a particular strength of the study. To our knowledge, this is the first study in Bangladesh to assess immunization related facility readiness. However, since the study involved health facilities from two purposively selected administrative divisions (one high performing and one low performing) of Bangladesh, interpretation of the findings should be carried out cautiously and might not be representative to the overall health system of Bangladesh as well as to that of other low-middle income countries. Again, we assessed only selected tracer indicators for general and immunization service readiness. Since the sample size calculation was not aimed to find out the differences between the divisions or various levels of health facilities, a relatively small sample size restricted us to perform statistical tests to distinguish readiness between high and low performing areas.

## Conclusions

Our study findings reported moderate to high levels of general service readiness for health facilities at different levels, except for pharmacies. Facilities at different levels suffered from lack of readiness in various aspects, but most notably in diagnostic capacity domain. No appreciable difference was observed between high and low performing divisions. Child immunization specific readiness was much higher among the facilities with vaccine storage capacity than that of their counterparts. Newly introduced PCV and IPV immunization lacked in funding and resources for training. Information provided in this study would help generating the evidence base to better inform the policymakers and related stakeholders in order to ensure equitable access and improve overall population health outcomes.

## Abbreviations

ABCE: Access, Bottlenecks, Costs and Equity
BCG: Bacillus Calmette-Guérin
CC: Community Clinics
DGHS: Directorate General of Health Services
EPI: Expanded Programme on Immunization
GPEI: Global Polio Eradication Initiative
icddr,b: International Centre for Diarrhoeal Disease Research, Bangladesh
IHME: Institute for Health Metrics and Evaluation
IPV: Inactivated Polio Vaccine
IRB: Institutional Review Board
MDG: Millennium Development Goal
MMR: Maternal Mortality Ratio
NGO: Non-government Organizations
NIPORT: National Institute of Population Research and Training
OPV: Oral Polio Vaccine
PCV: Pneumococcal Conjugate Vaccine
RD: Rural Dispensaries
SARA: Service Availability and Readiness Assessment
UH&FWC: Union Health and Family Welfare Center
UHC: Upazila Health Complexes
WHO: World Health Organization
